# A Rare Encounter: Incidental Ectopic Origin of the Right Pulmonary Artery in an Adult

**DOI:** 10.1055/a-2572-4238

**Published:** 2025-04-29

**Authors:** Rupali Jain, Maruti Kumaran, Achala Donuru

**Affiliations:** 1 Division of Cardiothoracic Radiology, Department of Radiology, Hospitals of University of Pennsylvania, Philadelphia, Pennsylvania; 2 Department of Radiology, Temple University Health System, Philadelphia, Pennsylvania

**Keywords:** ectopic right pulmonary artery, computed tomography angiography, adult presentation

## Abstract

Ectopic origin of the right pulmonary artery (RPA) from the aorta is a rare congenital anomaly typically found in infants. We report an adult female presenting with shortness of breath diagnosed incidentally with ectopic RPA via computed tomography angiography. This case underscores the need to consider rare congenital anomalies in adults presenting with unexplained pulmonary symptoms.

A 28-year-old woman presented for evaluation of shortness of breath. She reported feeling like a normal child while growing up. Physical examination revealed tachycardia, and her oxygen saturation was 80% on room air. Chest radiography demonstrated cardiomegaly, an enlarged pulmonary artery, and a mass-like opacity in the right hemithorax with a well-demarcated lateral margin, broad-based toward the mediastinum with hilum convergence sign ( Fig. 1 ).

**Fig. 1:**
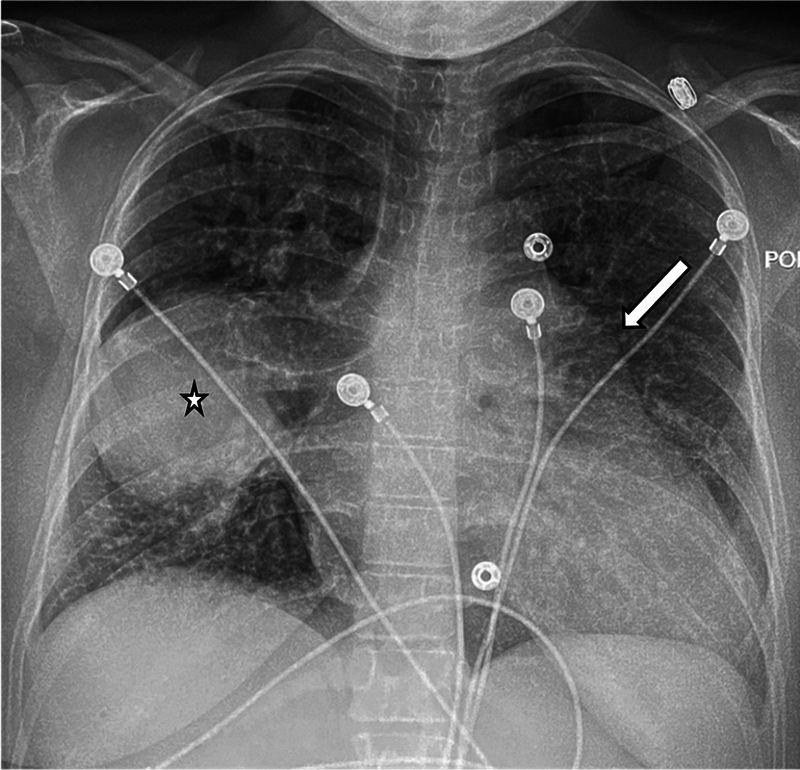
Frontal chest radiograph shows cardiomegaly, enlarged pulmonary artery (arrow), and a mass-like opacity in the right hemithorax (star) with well-demarcated lateral margin, broad-based toward the mediastinum with hilum convergence sign.

Computed tomography angiography (CTA) revealed the right pulmonary artery (RPA) arising from the aorta, whereas the left pulmonary artery originated from the main pulmonary trunk. A patent ductus arteriosus (PDA) was also present, connecting the aorta to the left pulmonary artery. The RPA was significantly enlarged with an eccentric thrombus and peripheral calcification, suggesting chronic thrombosis ( Figs. 2 , 3 , 4 , 5 ). Pulmonary venous drainage and aortic arch was normal.

**Fig. 2:**
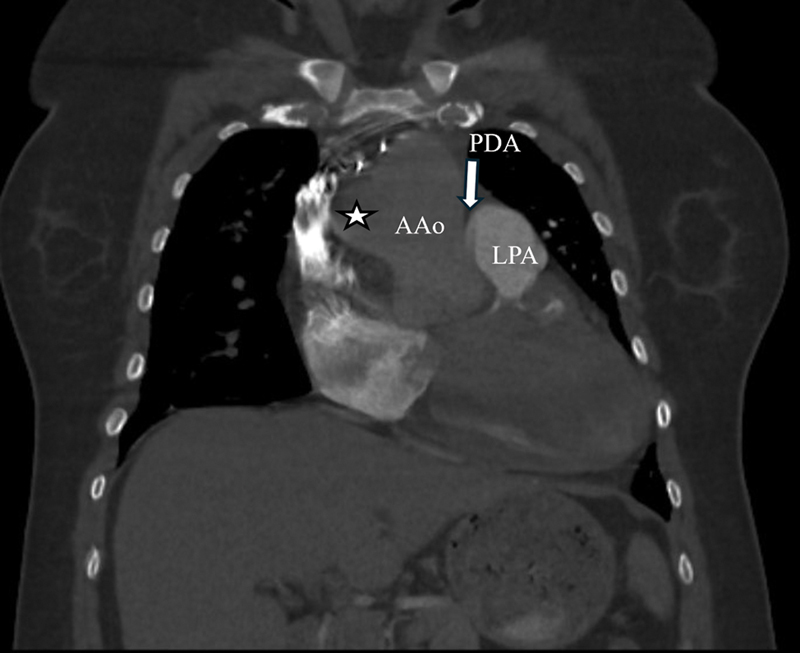
Early phase coronal computed tomography angiography (CTA) shows anomalous origin of the right pulmonary artery (RPA, star) from the ascending aorta (AAo). The left pulmonary artery (LPA) is better opacified than the aorta. Also noted is the patent ductus arteriosus (PDA, arrow).

**Fig. 3:**
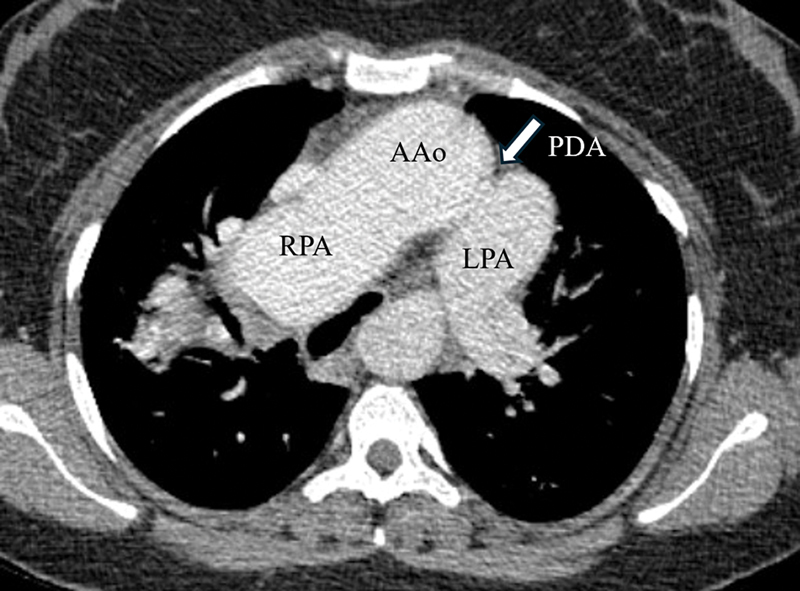
Delayed phase axial computed tomography angiography (CTA) shows anomalous origin of the right pulmonary artery (RPA) from the ascending aorta (AAo). The main pulmonary artery does not bifurcate but continues directly as the left pulmonary artery (LPA). Also noted is the patent ductus arteriosus (PDA, arrow).

**Fig. 4:**
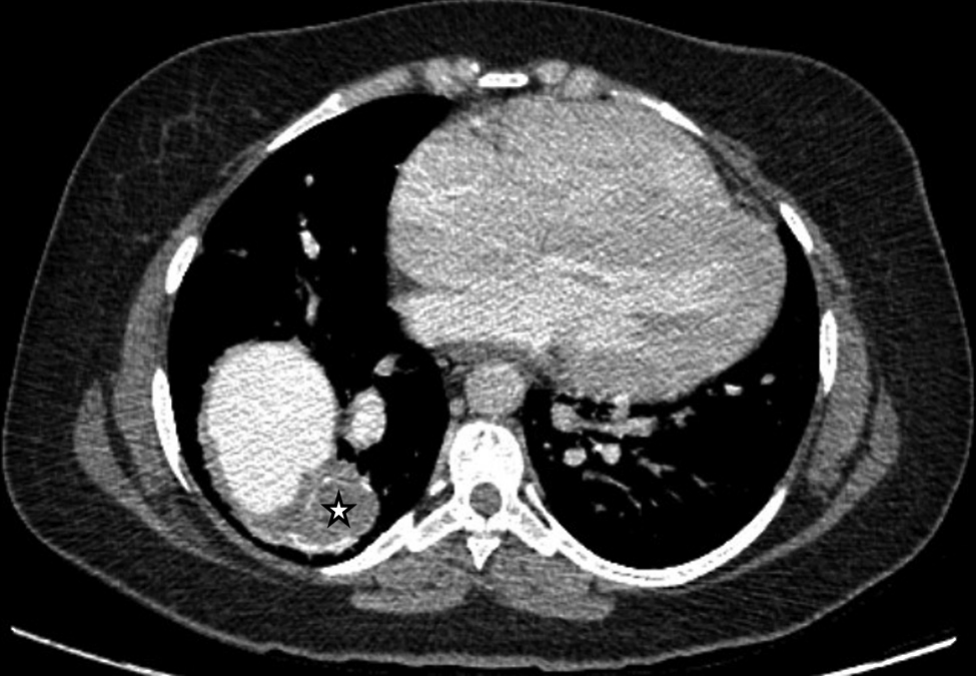
Delayed phase axial computed tomography angiography (CTA) shows a partially thrombosed right lower lobe pulmonary artery with peripheral calcification (star). This corresponds to the mass noted on the chest radiograph.

**Fig. 5:**
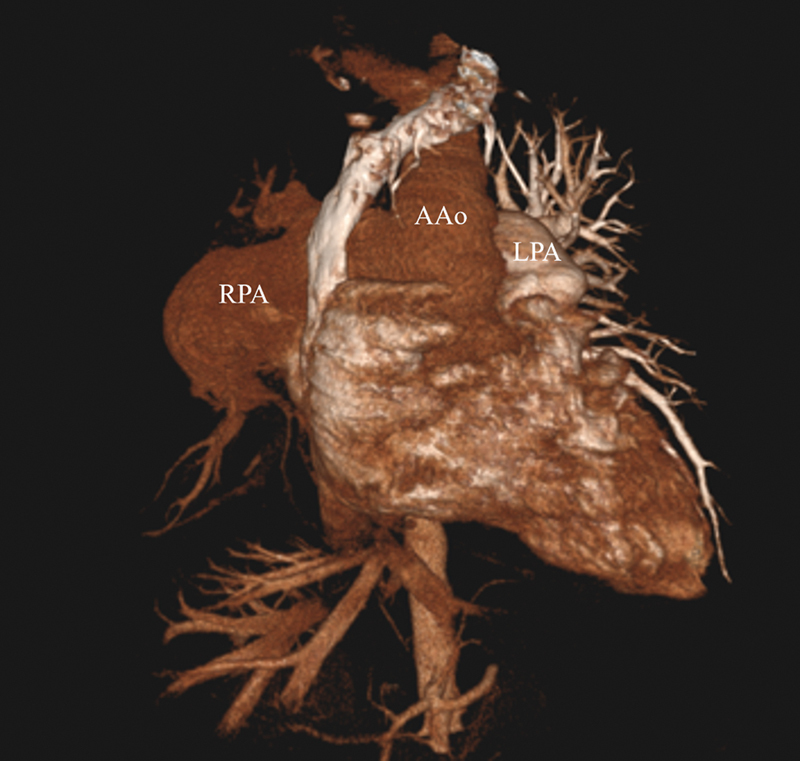
Cinematic rendering from computed tomography angiography (CTA) shows the abnormal right pulmonary artery (RPA) arising from the right lateral wall of the ascending aorta (AAo).

Further evaluation identified a unicommissural aortic valve with aortic stenosis, PDA with bidirectional shunting (right-to-left in systole, left-to-right in diastole), and elevated pulmonary artery pressure. The patient is currently on regular follow-up and symptomatically stable on Tadalafil and Ambrisentan; however, surgical intervention may be necessary if symptoms worsen.

Ectopic origin of the RPA from the aorta is exceedingly rare, with a reported prevalence of 0.33% in patients with known congenital cardiac anomalies. It typically presents in childhood, and early recognition with surgical correction is crucial for survival, as mortality rates reach 30% within the first 3 months without intervention. Our patient's presentation in adulthood is highly unusual. The ectopic pulmonary artery most commonly supplies the right lung and is often associated with a PDA. Untreated patients usually succumb during the first year of life due to terminal pneumonia and/or congestive heart failure. CTA effectively demonstrates this congenital defect. Cardiac magnetic resonance imaging can provide further insights into flow dynamics and velocity gradients, particularly valuable in rare adult cases.

